# The Freeze-Drying of Pharmaceuticals in Vials Nested in a Rack System—Part II: Primary Drying Behaviour

**DOI:** 10.3390/pharmaceutics15112570

**Published:** 2023-11-02

**Authors:** Fiora Artusio, Marco Adami, Antonello A. Barresi, Davide Fissore, Maria Chiara Frare, Claudia I. Udrescu, Roberto Pisano

**Affiliations:** 1 Department of Applied Science and Technology, Politecnico di Torino, 24 Corso Duca degli Abruzzi, 10129 Torino, Italy; fiora.artusio@polito.it (F.A.); davide.fissore@polito.it (D.F.);; 2 Independent Consultant, 20100 Milano, Italy; 3 Stevanato Group, 17 Via Molinella, 35017 Piombino Dese, Italy; mariachiara.frare@stevanatogroup.com

**Keywords:** freeze-drying, primary drying, sublimation flux, heat transfer, sucrose, rack system, mannitol

## Abstract

The freeze-drying of biopharmaceuticals is a common strategy to extend their shelf-life and facilitate the distribution of therapeutics. The drying phase is the most demanding one in terms of energy consumption and determines the overall process time. Our previous work showed how the loading configuration can impact freezing. This paper focuses on primary drying by comparing the thermal behaviour of vials loaded in direct contact with the shelf or nested in a rack system. The overall heat transfer coefficient from the apparatus to the product was evaluated at different chamber pressures (5–30 Pa) and shelf temperatures (from −10 °C to +30 °C), and in the case of various vial positions (central, semi-border, and border vials). Because of the suspended configuration, the heat transfer coefficient was less affected by chamber pressure in vials nested in a rack system. The two loading configurations displayed comparable heat transfer efficiency below 10 Pa. For higher chamber pressure, the heat transfer coefficients of nested vials were lower than those of vials in direct contact with the shelf. Nevertheless, the rack system was beneficial for reducing the inter-vial variability as it promoted higher uniformity in the heat transfer coefficients of central vials. Eventually, thermal image analyses highlighted limited temperature differences between the vials and the rack system.

## 1. Introduction

Freeze-drying is commonly used to extend the shelf-life and ease the handling of biopharmaceuticals [1,2]. The process is often carried out in vials to guarantee ready-to-reconstitute unit doses, avoiding dosing errors and contamination risks. Since freeze-drying is time- and energy-consuming, alternative drying methods have been proposed, such as spray drying or supercritical drying [3,4]. However, thermal and atomisation stresses and the requirement of special equipment still limit their industrial application.

Careful design of the freeze-drying process is thus essential to reduce downtime and shorten process time, guaranteeing safe operating conditions. Cycle optimisation is based on the knowledge of critical formulation properties, such as the collapse temperature, the type of excipients used, and the heat transfer efficiency between the equipment and the primary packaging [5,6,7]. The latter aspect determines the amount of heat that can be exchanged with the product during the freezing and drying steps. This aspect is particularly relevant during primary drying when the frozen product undergoes ice sublimation. The heat transfer efficiency and processing conditions, i.e., shelf temperature and chamber pressure, impact the product temperature, which must be maintained below a specific threshold value to avoid product collapse [8]. Thus, quantifying heat transfer during primary drying is of utmost importance in designing optimal freeze-drying cycles.

The most common primary packaging in the pharmaceutical freeze-drying industry is represented by glass vials, which can be either tubing or, less commonly, moulded vials [9]. Unlike other parenteral products, the primary packaging of freeze-dried pharmaceuticals does not only serve storage purposes but is directly involved in the freeze-drying process. The thermal performance of the product container determines the amount of heat that can be transferred from the equipment to the product. Heat can be supplied to the product by means of three main mechanisms during primary drying: direct contact between the shelf and the vial bottom, conduction through the gas trapped between the shelf and the vial bottom, and radiation coming from the chamber walls, the door (which can be several degrees warmer than the shelf set-point), and the upper shelf. The relative weight of each mechanism can strongly vary with the vial position within the batch and the loading configuration of the vials. Regarding the vial position, edge vials, i.e., the more external vials of the batch facing the chamber walls, experience higher heat transfer than central vials due to the higher amount of heat received by radiation from the walls and through gas conduction because of their different packing density compared to border and central vials [10,11]. Furthermore, in some cases, a metallic frame may be used for vials loading, contributing to the heat flux to the side vials by radiation and conduction in the contact points [12,13,14].

Regarding the loading configuration, it has been reported that radiation accounts for approximately 25–40% of the heat power supplied to the product during drying when border vials are directly loaded on the shelf, whereas it increases to approximately 95% when vials are suspended over the shelf [15].

Even different vial manufacturing techniques and glass compositions can impact heat transfer [16], e.g., because of variations in the vial bottom geometry and the container heat conductivity. The vial bottom geometry determines the extent of the contact area with the shelf and the thickness of the gas gap, and it strongly affects heat transfer. Scutellà et al. [17] reported that even the geometrical variability within the same batch of vials could affect the product temperature, resulting in significant vial-to-vial heterogeneity during a freeze-drying cycle. The effect was more pronounced, i.e., up to 2.2 °C variability in the product temperature of central vials, at low chamber pressure due to the increased contribution of contact conduction.

Recently, vial-loading devices made of aluminium have been studied to facilitate the loading and positioning of high-throughput vials [18] or Polymerase Chain Reaction (PCR) vials [19]. On a lab-scale unit, 96-well plates hosting high-throughput vials were employed to test a large number of formulations with low amounts of active ingredients to enhance formulation screening [18]. Using a well plate substantially impacted heat transfer, resulting in predicted product temperatures 8 °C higher than serum vials. When vials are inserted in 96-well plates, the contribution of heat transfer from the walls of the container system is significant. This feature can impact the ice sublimation interface, leading to a cone structure during sublimation tests on pure ice. Furthermore, 96-well freeze-drying has been performed in aluminium blocks to obtain more uniform heat transfer and reduce the edge effects observed in plastic plates [13].

The use of secondary packaging for hosting vials has gained interest for the industrial freeze-drying of unit doses. Currently, vials are loaded on trays and kept in place with the help of side rails or metallic frames [20]. The use of a rack minimises mechanical impacts that are often a source of vial breakage during vial transportation and handling, reduces contamination risks by simplifying the loading under sterile conditions, and can shorten loading and unloading downtime. Rack systems can potentially ensure reduced vial-to-vial heterogeneity, provided that good contact between the vials and holder is ensured [21].

In our recent work [22], we showed how the use of a rack system alters the freezing conditions and, hence, the frozen product morphology. This paper extends this analysis to primary drying; specifically, the resistance to heat transfer from the freeze-dryer to the product under lyophilisation was studied varying the loading configuration and primary drying conditions. The comparison of the heat transfer coefficients of vials in direct contact with the shelf and nested in the rack system aims to highlight some aspects that must be considered when transferring existing freeze-drying cycles, which are developed for the direct contact configuration, to the nested configuration. To the best of our knowledge, this is the first study on the impact of a commercial rack for vials on the freeze-drying process conditions.

## 2. Materials and Methods

### 2.1. Materials

All the tests were carried out in 4-cc tubing vials (2R ISO, Stevanato Group, Piombino Dese, Italy). Deionised water was used for the gravimetric tests, whereas the freeze-drying cycles were performed using 5 wt% mannitol or 5 wt% sucrose (Merck) solutions. All of the reagents were of analytical grade and used as received.

An alveolar secondary packaging (SG EZ-fill^®^ Nest, Stevanato Group, Piombino Dese, Italy) was used to host 100 tubing vials. In this configuration, vials were raised to approximately 1 mm above the shelf in a hexagonal arrangement.

### 2.2. Experimental Set-Up

All of the tests were performed in a lab-scale freeze-dryer (Revo, Millrock Technology, Kingston, New York, NY, USA). Vials were loaded considering two configurations. Vials were either directly placed on the shelf in a hexagonal arrangement or loaded in the rack system. The temperature of some reference vials was monitored via T-type miniature thermocouples (Tersid, Milano, Italy). For vials loaded in direct contact with the shelf, each test involved 210 vials. The sensors were placed in the bottom centre of vials representative of central (5), semi-border (3), and border (2) vials to record the thermal evolution in each group of vials. Semi-border vials refer to vials placed in the second row moving from the edge to the central vials. For nested vials, two nests hosting 100 vials each (200 vials in total) were used for each test. The sensors were placed inside the central (4), semi-border (2), and border vials (2) of each nest. In all tests, the average temperature evolution of each class of vials was considered to calculate heat fluxes and heat transfer coefficients. Monitoring the thermal evolution of each class of vials, instead of just recording the temperature of central vials, reduces the risk of introducing a systematic bias since the thermal evolution of central vials is not representative of the entire batch [23]. The temperature of the heat transfer fluid was monitored through Pt100 sensors.

All the vials were filled with 2 deionised water to perform the gravimetric tests. Vials were individually weighed before and after each test. In a typical test, vials were loaded into the freeze-dryer chamber and cooled at −1 °C/min to −45 °C. Then, ice sublimation was carried out for approximately 4 h. The list of investigated chamber pressures (*P_C_*) and shelf temperatures (*T_S_*) is reported in Table 1.

### 2.3. Determination of the Heat Transfer Coefficient

The (overall) heat transfer coefficient between the equipment and the product was obtained considering that heat is exchanged via different mechanisms depending on the loading configuration. The contribution of each mechanism (radiation, conduction through the gas, direct contact) is also a function of the vial position within the batch. Vials were grouped as central, semi-border, and border vials.

The heat transfer coefficient (*K_v_*) was determined for each vial of the batch by a gravimetric test. Knowing the weight loss after the sublimation phase (Δm) and the duration of the sublimation phase (Δt), *K_v_* was calculated as:

(1) $$K_{v}=\frac{\Delta m\Delta H_{s}}{S\int_{0}^{\Delta t}\left(T_{S}-T_{B}\right)dt}$$

where $\Delta H_{s}$ is the ice sublimation enthalpy, *S* is the internal cross-sectional area of the vial, and *T_S_* and *T_B_* are the shelf and product temperatures, respectively. The average evolution of *T_B_* of the reference group of vials was considered for the calculation, whereas Δm was individually measured for each vial. The uncertainty of *K_v_*, correlated to the product temperature measurement, was discussed in [24]. The heat flux supplied to each vial (*J_q_*) is given by:

(2) $$J_{q}={\Delta H}_{s}\frac{\Delta m}{S\Delta t}$$

Corner vials were excluded from the calculation of the heat transfer coefficient as their number is not statistically relevant and because of the high variability in the heat received by each corner vial. Nevertheless, even if the thermal evolution of corner vials had not been recorded, it was still possible to calculate the sublimation rate of corner vials knowing Δm of each corner vial and the sublimation time.

### 2.4. Analytical description of K_v_

*K_v_* can be described as a function of chamber pressure in the form of:

(3) $$K_{v}=A+\frac{BP_{c}}{1+CP_{c}}$$

where *A*, *B*, and *C* coefficients account for the different contributions to heat transfer. In particular, *A* accounts for heat transfer through radiation and direct contact between the shelf and the vial. *B* and *C* consider the heat exchanged by conduction through the gas. *A* is a function of the vial position, whereas *B* and *C* only depend on the loading configuration and are the same for all the vials. A detailed derivation of Equation (3) is given in [12].

### 2.5. Thermal Imaging Camera Analyses

**Figure 1:**
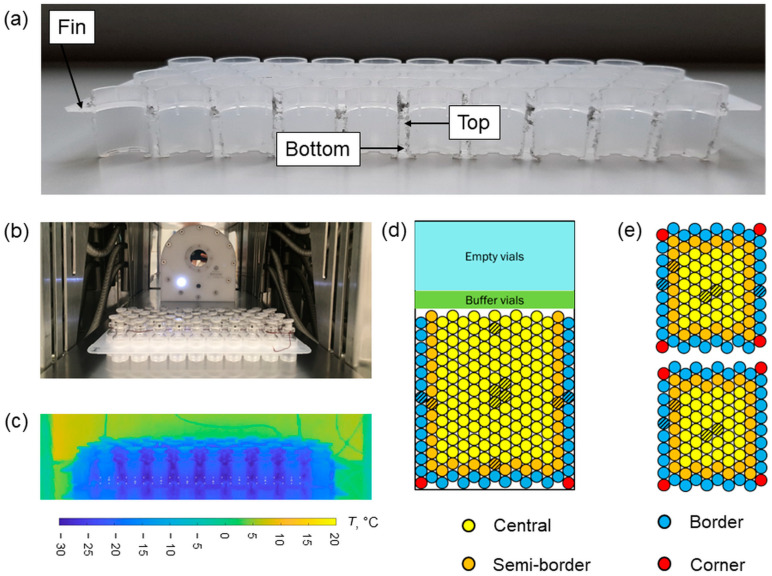
(**a**) Schematic of the position of points investigated with the thermal imaging camera on the cross-section of the rack system. (**b**) Experimental set-up used for thermal imaging investigation. (**c**) Thermal image of the vials nested in the rack system as recorded with the thermal imaging camera. Schematics of the loading of vials in (**d**) direct contact with the shelf and (**e**) nested in the rack system. Dashed positions refer to vials containing thermocouples.

The temperature distribution of the rack system was also investigated through a thermal imaging camera placed in a thermally insulating enclosure (FLIR thermal camera, model A35, IMC Services s.r.l., Mascalucia (CT), Italy). The rack system was cut along the axial direction to image the cross-section. Vials were filled with 2 mL of deionised water. The emissivity of the rack system was first measured following the ISO 18434-1 guideline [25]. A mean value of 0.866 (in the range from −50 °C to 30 °C) was obtained using the contact method. The operating conditions of Tests #2 and 4 were used (freezing at −45 °C, primary drying at −10 °C, and 10 or 30 Pa). Specific points at the top, bottom, and on the fin of the rack system were monitored, as shown in Figure 1a, to investigate axial thermal gradients. In addition, the mean minimum and maximum temperatures of the central vials were monitored, corresponding to the temperature of the sublimating interface and the maximum temperature of the axial temperature profile of the vial, respectively. Also, the temperature of the vial bottom was extracted. The minimum, maximum, and bottom temperatures were averaged considering the eight central vials (the two external vials were excluded to avoid edge effects). The experimental set-up and a representative thermal image of the vials nested in the rack system are reported in Figure 1b,c.

### 2.6. Freeze-Drying Cycles

Vials were filled with 2 mL of either 5 wt% mannitol or 5 wt% sucrose solutions. Vials were either directly loaded on the shelf or nested in the rack system. Thermocouples were placed inside vials in central positions in both configurations. Vials containing mannitol solutions were frozen at −40 °C, annealed at −20 °C (2 h), and cooled again to −40 °C. During primary drying, the chamber pressure and shelf temperature were 10 Pa and −10 °C. Secondary drying was performed at +40 °C for 8 h.

A second freeze-drying cycle involving vials filled with 2 mL of 5 wt% sucrose solutions was performed and monitored via a thermal imaging camera. Vials were loaded in direct contact with the shelf or in the rack system. For this test, vials were frozen at −45 °C and dried at 10 Pa and −25 °C.

## 3. Results

### 3.1. Vials in Direct Contact with the Shelf

**Figure 2:**
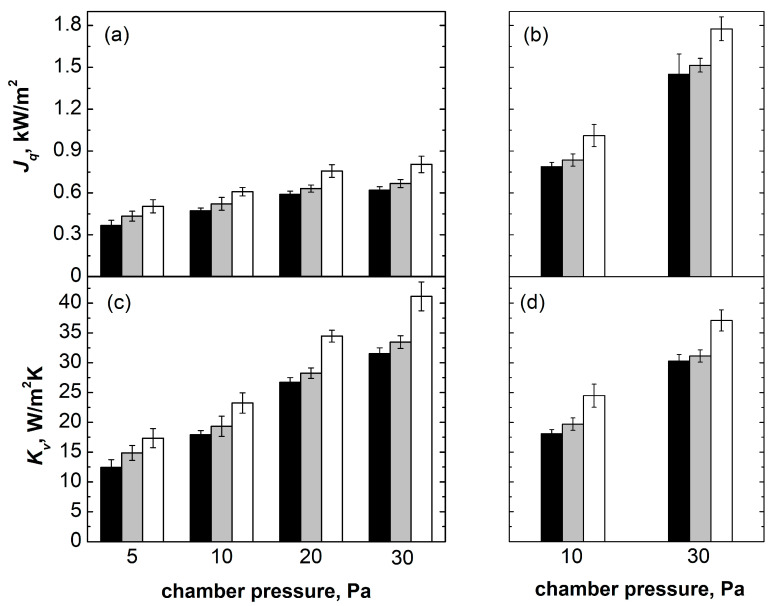
*J_q_* and *K_v_* as a function of *P_C_* and *T_S_* for (■) central, (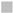) semi-border, and (☐) border vials in direct contact with the shelf. Data reported in (**a**) and (**c**) refer to *T_S_* = −10 °C for various chamber pressures; data reported in (**b**) and (**d**) refer to *T_S_* = +10 °C for *P_C_* = 10 Pa and *T_S_* = +30 °C for *P_c_* = 30 Pa.

The heat flux (*J_q_*) and the overall heat transfer coefficient (*K_v_*) for central, semi-border, and border vials directly resting on the shelf are shown in Figure 2 as a function of *P_C_* and *T_S_*. Considering *T_S_* = −10 °C, *K_v_* increased with *P_C_* for all of the groups of vials because of the increasing contribution of heat exchanged by conduction through the gas trapped between the shelf and the vial bottom (see Figure 2c). *K_v_* increased moving from central to border vials and was the highest for border vials. This behaviour resulted from the vial position-dependent contribution of heat exchange by radiation, being maximum for vials directly facing the chamber walls.

A similar trend for the different groups of vials was also observed at *T_S_* = +10 °C (*P_C_* = 10 Pa) and *T_S_* = +30 °C (*P_C_* = 30 Pa); see Figure 2b,d. *J_q_* values were significantly higher for all groups of vials, especially at *T_S_* = +30 °C and *P_C_* = 30 Pa because of the larger driving force for ice sublimation. Nevertheless, *K_v_* values overlapped with those obtained at *T_S_* = −10 °C, suggesting a negligible dependence of *K_v_* vs. *T_S_*, in agreement with previous studies [26].

### 3.2. Vials Nested in a Rack System

**Figure 3:**
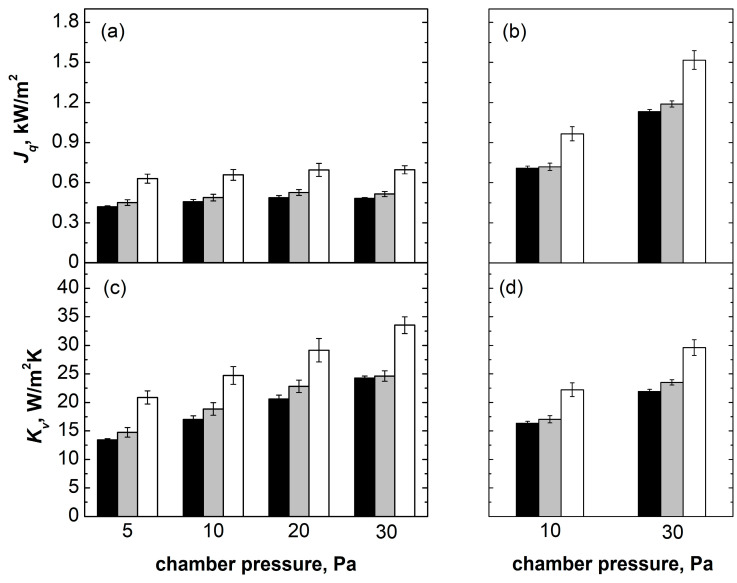
*J_q_* and *K_v_* as a function of *P_c_* and *T_s_* for (■) central, (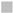) semi-border, and (☐) border vials nested in the rack system. Data reported in (**a**) and (**c**) refer to *T_S_* = −10 °C for various chamber pressures; data reported in (**b**) and (**d**) refer to *T_S_* = +10 °C for *P_C_* = 10 Pa and *T_S_* = +30 °C for *P_C_* = 30 Pa.

Figure 3 reports the values of *J_q_* and *K_v_* obtained for different groups of vials nested in the rack system as a function of *P_C_* and *T_S_*. *K_v_* increased as a function of *P_C_* for all three groups of nested vials. *K_v_* also increased moving from central to border vials. As obtained for vials in contact with the shelf, *K_v_* had a negligible dependence on *T_S_*.

At low chamber pressure, i.e., 5 and 10 Pa, *K_v_* values of central and semi-border vials were comparable to those obtained for vials directly resting on the shelf. At higher chamber pressures, the *K_v_* values of all three groups of vials were smaller than in the resting configuration. It has been reported that the capacity of the exchanging heat of vials in a suspended configuration is lower than that of vials directly resting on the shelf because of the absence of direct contact heat transfer and the reduced contribution of gas conduction. This phenomenon becomes particularly evident when the distance between the vial bottom and the shelf is sufficiently large (approximately 3 mm) [15]. Considering nested vials, this distance was approximately 1 mm. The dependence of *K_v_* on *P*_c_ resulted from the reduced but still not negligible conduction through the rarefied gas for vials hosted in the rack system.

Interestingly, the *K_v_* of border vials was approximately 40–55% higher than central vials for the nested configuration, whereas it was only approximately 30–40% higher for the direct contact configuration. This result suggests that border vials in the rack system are not able to transfer heat to adjacent vials as efficiently as vials in direct contact with each other because of the presence of the separating material.

**Figure 4:**
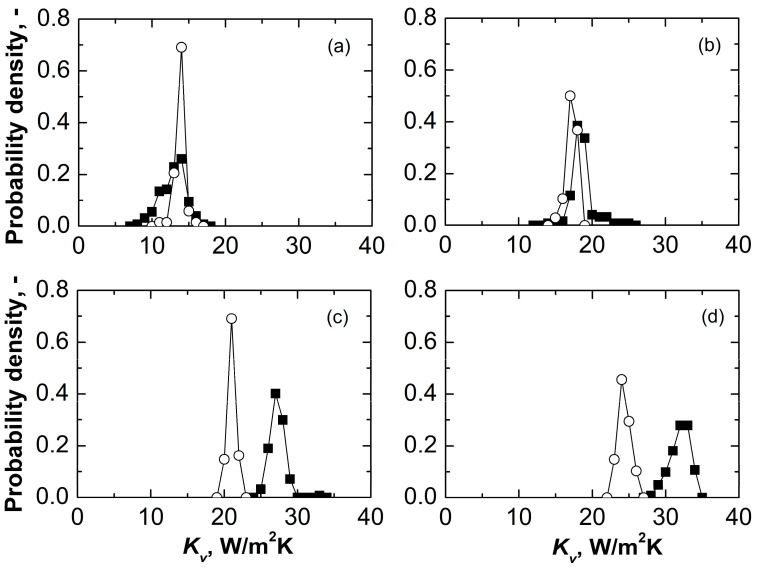
*K_v_* probability density distributions obtained for central vials in direct contact with the shelf (black symbols) and nested in a rack system (white symbols). Figure panels refer to *P_C_* = (**a**) 5, (**b**) 10, (**c**) 20, and (**d**) 30 Pa.

The *K_v_* of nested vials had approximately one order of magnitude lower standard deviations for central vials (0.20 Wm^−2^K^−1^ against 1.29 Wm^−2^K^−1^ at 5 Pa). The increased uniformity of *K_v_* of nested vials was confirmed by the sharper *K_v_* distributions obtained for central vials at various pressures, as shown in Figure 4a–d. This beneficial effect on inter-vial heat transfer variability was attributed to the absence of contact between the shelf and the vial bottom. The shelf is a heterogeneous heat source due to its imperfect planarity and thermal gradients in the heat transfer fluid [17].

**Figure 5:**
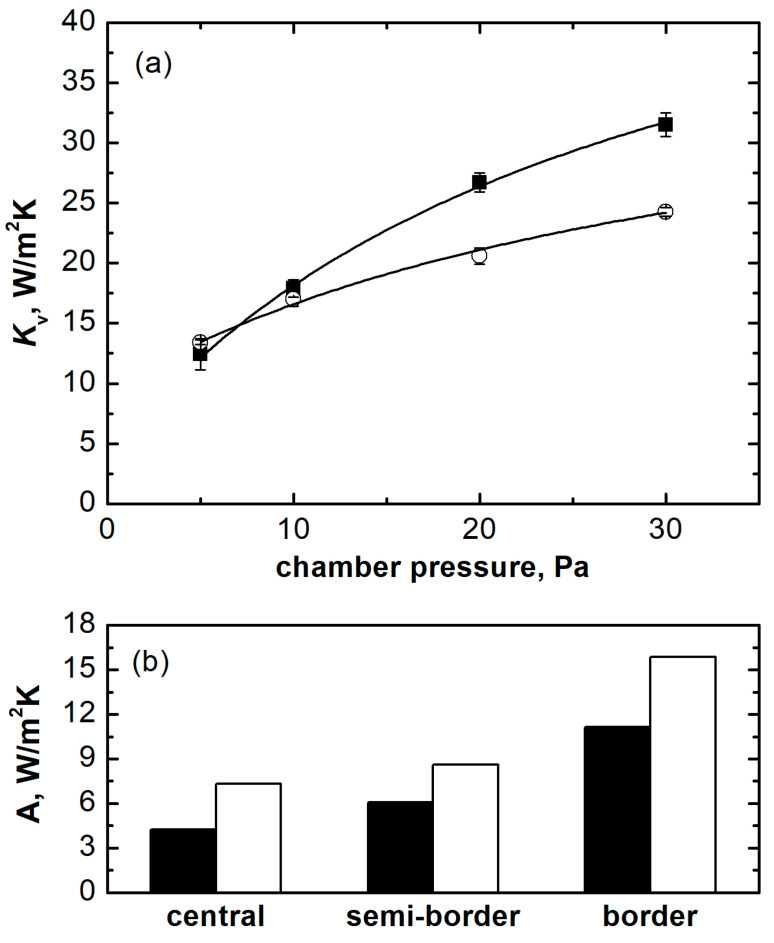
(**a**) Mathematical model predictions of *K_v_* as a function of *P_C_* for central vials, and (**b**) comparison of the *A* parameter in Equation (3) for central, semi-border, and border vials in the two loading configurations. Black symbols/bars refer to vials in direct contact with the shelf, white symbols/bars refer to vials nested in the rack system.

The *K*_v_ experimental data were used to derive the parameters of the mathematical model presented in Equation (3) considering the two loading configurations, as shown in Figure 5a. The *K_v_* of nested vials displayed a less marked dependence on *P_C_* compared to vials in direct contact with the shelf. This behaviour suggests a comparable heat exchange efficiency between the two loading configurations at low pressure (*P_C_* < 10 Pa) and a less efficient heat transfer efficiency for nested vials at higher pressures (*P_C_* > 10 Pa).

The parameters of the mathematical model for central, semi-border, and border vials are reported in Table 2. Parameters *B* and *C* of the model are the same for all the groups of vials as they only depend on the loading configuration. A comparison between parameter *A* obtained for vials in different positions of the two loading configurations is reported in Figure 5b. The increasing contribution of radiation from the chamber walls moving from central to border vials was responsible for the corresponding increase in the value of *A*. For all groups of vials, *A* was consistently higher for vials nested in the rack system. Because of the loading device geometry, nested vials are less packed than vials in direct contact with the shelf. Thus, the amount of radiative heat received by all the vials of the batch is higher compared to the direct contact configuration, because of different view factors. The increase in heat exchanged by radiation compensates for the loss of heat exchange by direct contact and the reduced contribution of heat exchanged by gas conduction. In addition, border vials are characterised by lower packing density compared to central vials, resulting in a factor that also contributes to inhomogeneous ice sublimation rates in the batch, as recently reported [10,11]. The heterogeneity in the heat received by the different groups of vials during primary drying results in marked differences in the sublimation rates. For example, at 10 Pa, the sublimation rate of central and corner vials in direct contact with the shelf was 1.78 × 10^−4^ and 2.67 × 10^−4^ kg/sm^2^, respectively. The sublimation rate of central and corner vials nested in the rack system was 1.68 × 10^−4^ and 2.87 × 10^−4^ kg/sm^2^, respectively. This result highlights that corner vials nested in the rack system are more susceptible to fast drying compared to corner vials loaded in direct contact with the shelf because of the higher amount of heat received and available for sublimation.

### 3.3. Thermal Imaging Camera Investigation

**Figure 6:**
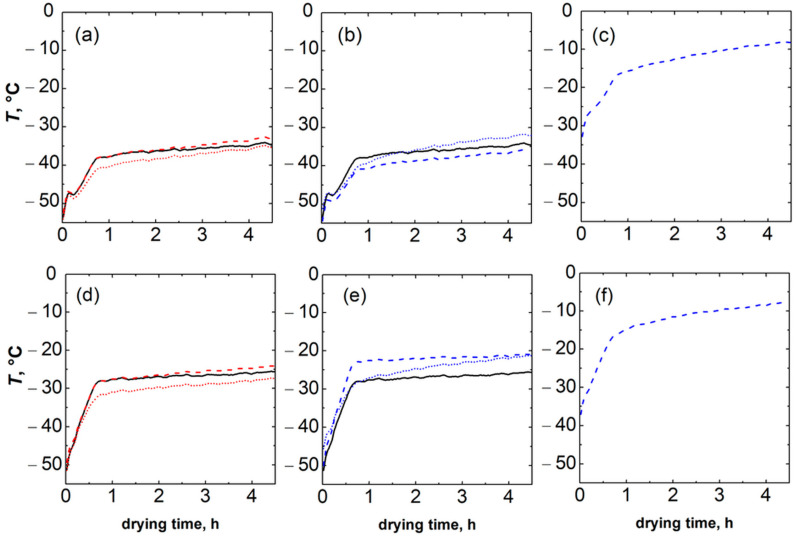
IR profiles obtained during the ice sublimation phase of (**a**) the average temperature of the sublimation interface (red dotted line) and average maximum temperature of the thermal axial profile in the vial (red dashed line) and average temperature of the vial bottom (black line), (**b**) the top (blue dotted line) and bottom (blue dashed line) areas (see Figure 1a for reference) of the rack system and vial bottom (black line), and (**c**) the rack system fin at 10 Pa. (**d**–**f**) Analogous thermal profiles obtained at 30 Pa.

A thermal imaging camera was used to better understand the impact of the rack system on the heat transfer between the equipment and the vials. Infrared thermography allows for non-invasive monitoring of freeze-drying processes [27]. Specific points were considered, i.e., the average temperature of the sublimation interface (*T_min_*), the average maximum temperature of the thermal axial profile in the vial (*T_max_*), the average temperature of the vial bottom (*T_B_*), the nest points at the top and bottom, and the nest fin. For all of the tests, water was frozen at −45 °C for 2 h while primary drying was performed at −10 °C and 10 or 30 Pa. The representative thermal evolution profiles reported in Figure 6 refer to the ice sublimation step and highlight remarkable axial thermal gradients in the rack system (a difference of up to 5 °C between top and bottom points). This behaviour was explained considering that the bottom points are close to the portion of the vial in contact with the product, which is sublimating and subtracting heat. At both chamber pressures, it appears that, at the beginning of the sublimation phase, the thermal profiles of the rack system and the vials were overlapping due to the small radiative heat contribution and the slow process dynamics. However, when ice sublimation is established, the cooling effect due to the phase change affects both the vials and the rack system, resulting in a marked reduction in the slope of the temperature profiles of both the vials and the rack system. Thus, the thermal evolution of the rack system during primary drying follows that of the vials.

Interestingly, at 30 Pa, the temperature of the bottom points of the rack system was higher than the top points (see Figure 6e).

This controversial behaviour highlights some peculiarities of the heat transfer mechanisms when using a rack system. The temperature of the rack system results from the balance between the heat transferred from the equipment to the rack system and the heat exchanged with the vials during sublimation. The heat transfer from the equipment to the rack system results from the contribution of radiation (pressure independent) and gas conduction (pressure dependent). At low pressure (10 Pa), the contribution of gas conduction is less relevant, and the top points of the rack system are hotter than the bottom points because of the heat received by radiation. As chamber pressure affects the driving force for ice sublimation and the weight of the various heat transfer mechanisms among the equipment, the rack system, and the vials, product temperature is also accordingly affected. At higher pressures, i.e., 30 Pa, the product and the rack system temperatures were higher, as shown in Figure 6e.

Overall, the temperature of the rack system is the result of the heat transferred from the shelf, the radiation from the chamber walls, and the exchange with the product. The configuration used for the thermal imaging camera investigation is more affected by radiation compared to the standard one, as the rack system has been cut along the axial direction. Nevertheless, it can be concluded that the rack system adds a slight resistance to heat transfer to the vial. However, the temperature difference between the vial and the rack system is minimal, suggesting a negligible contribution of the sides of the rack system to the heat transferred to the product.

### 3.4. Primary Drying Behaviour

The impact of the loading configuration of vials on the primary drying behaviour was studied considering two model formulations, i.e., 5 wt% mannitol and 5 wt% sucrose, and different techniques.

**Figure 7:**
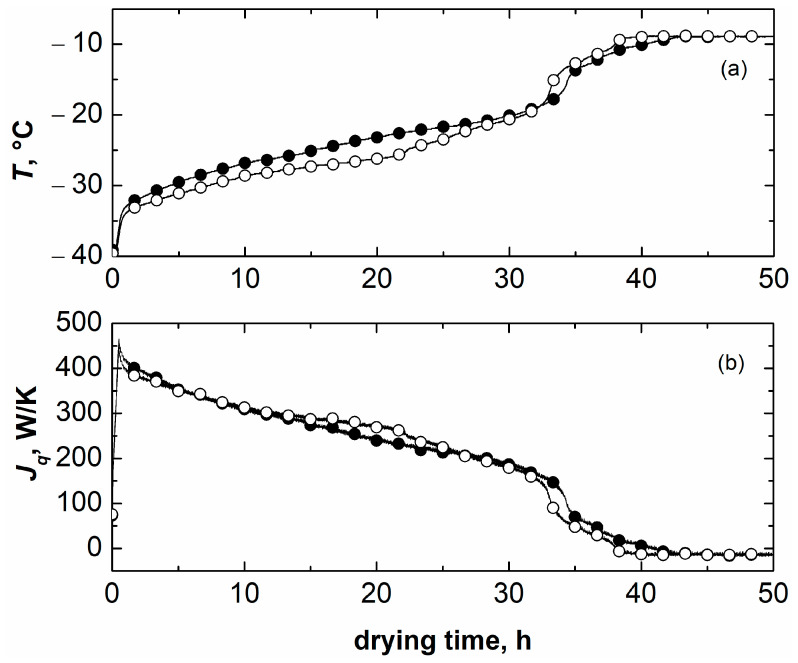
Evolution of (**a**) product temperature and (**b**) thermal heat flux *J_q_* inside central vials during primary drying as monitored through thermocouples. Black symbols refer to a vial placed in direct contact with the shelf, whereas white symbols refer to a nested vial. 5 wt% mannitol was used as a model product. Primary drying conditions were *T_S_* = −10 °C and *P_C_* = 10 Pa.

First, a freeze-drying cycle involving a 5 wt% mannitol solution was performed to compare the thermal behaviour of the product during primary drying by means of thermocouples. The use of thermocouples for measuring product temperature represents a reliable method by which to monitor the thermal evolution of a batch of vials. It has been reported that the insertion of thermocouples inside selected vials does not significantly alter, at least in non-GMP conditions, the cake structure, hence the resistance to vapor flow and the drying behaviour of the monitored product [28,29]. Thus, the presence of thermocouples does not compromise the reliability of the thermal evolution of the monitored vials, which can still be considered representative of the corresponding group of vials (central, semi-border, and border). The cycle was performed at 10 Pa since the two configurations have comparable *K*_v_ values, making it possible to identify (if any) the effects of the dried product morphology on product temperature. The thermal evolution of representative vials in direct contact with the shelf and nested in the rack system is shown in Figure 7a.

During the ice sublimation phase, the temperature of the product in the nested vials was approximately 2 °C lower compared to the direct contact configuration. This result was attributed to the interplay between the heat transfer efficiency and the morphology of the dried layer in the two configurations. Nested vials have smaller *K_v_* but lower resistance to vapour flow. Our previous work [22] showed that nested vials led to larger pore size, as a consequence of the tendency of these vials to nucleate at higher temperatures. As a result, the product temperature in nested vials is lower because of the lower resistance to vapor flow of the dried layer. When sublimation is established, the thermal heat flux supplied to the vials is slightly higher for nested vials; see Figure 7b. A more open structure is also beneficial for primary drying duration: nested vials completed drying in approximately 32 h, whereas vials loaded in direct contact with the shelf required approximately 34 h. Nevertheless, the differences in product temperature and drying time are not pronounced, since the thermal effects and the structure-related effects tend to compensate each other at 10 Pa.

**Figure 8:**
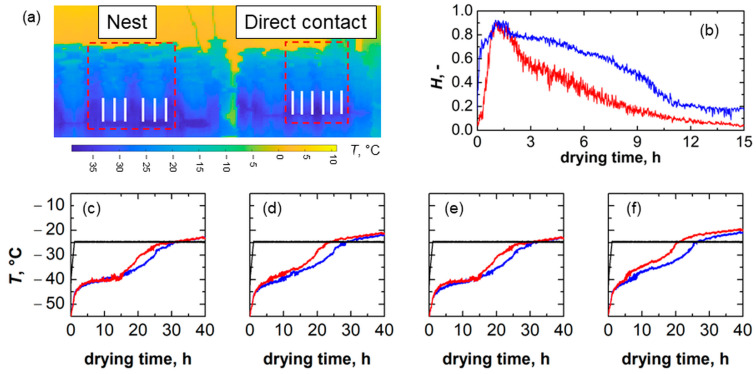
(**a**) Extraction points of vials nested in the rack system and in direct contact with the shelf. (**b**) Comparison between the evolution of the non-dimensional position of the minimum temperature of vials loaded in the rack system (blue curve) and in direct contact with the shelf (red curve). Evolution of (**c**) vial bottom, (**d**) average, (**e**) minimum, and (**f**) maximum temperature during primary drying. Red curves refer to vials in direct contact with the shelf, blue curves refer to vials nested in the rack system, and black curves refer to shelf temperature.

A second test was performed using a thermal imaging camera to monitor the temperature evolution of selected points of vials in direct contact with the shelf and nested in the rack system during the same freeze-drying cycle. A 5 wt% sucrose solution was used. The analysis focused on two central vials per configuration, as shown in Figure 8a (see the dashed perimeter). By tracking the non-dimensional position of the minimum vial temperature, *H*, which corresponds to the interface position (normalised with respect to the difference between the initial position of the minimum and maximum vial temperature) for each set of vials during primary drying, see Figure 8b, a difference in drying time was highlighted. The *H* of vials in direct contact with the shelf moves faster towards the bottom of the vial compared to nested vials. Such a behaviour was confirmed by the profiles of the bottom, average, minimum, and maximum temperatures of vials (Figure 8c–f). It appears that the product temperature in vials directly loaded on the shelf is higher than that of the product of nested vials.

Nevertheless, despite being a non-invasive temperature monitoring technique, infrared thermography results were obtained under conditions that differed from the previous freeze-drying test. As only first-row vials could be monitored with the experimental set-up used in this study, the relative contribution of the various heat transfer mechanisms for these vials could be significantly different from that of vials in the central position of a batch, especially as far as radiative heat is concerned. Nevertheless, the rack system still impacted the product behaviour during primary drying.

## 4. Conclusions

The impact of the loading configuration of vials on heat transfer during primary drying was investigated. Vials in direct contact with the shelf and loaded in a rack system displayed comparable heat transfer efficiency for chamber pressures below 10 Pa. At higher chamber pressure, vials loaded in the rack systems received less heat because of the reduced contribution of conduction through the gas and the absence of direct contact with the shelf. This aspect has to be taken into account when transferring existing freeze-drying protocols developed for resting vials to vials nested in the rack system so as to avoid cycle failures. The loading device has a limited impact on the resistance to heat transfer supplied to the vials, as shown by the thermal imaging camera investigation. Nevertheless, the distribution of *K_v_* within the batch showed reduced variability for vials nested in the rack system compared to vials in direct contact with the shelf, suggesting a more uniform heat transfer from the equipment to the product among the batch. Such an attribute is particularly interesting to reduce inter-vial variability and increase batch homogeneity. Moreover, compared to traditional bulk configuration, the rack system ensures the increased integrity of pre-sterilised vials during transportation and in buffering/in-feeding operations thanks to the separation of all containers in the secondary packaging. The risk of vial breakages potentially generated from glass-to-glass contact is effectively mitigated, as well as that of cosmetic issues and particle generation.

## Figures and Tables

**Table 1:** Chamber pressure (*P_c_*) and shelf temperature (*T_S_*) values used to perform gravimetric tests for the experimental determination of the heat transfer coefficient.

Test #	Chamber Pressure (*P_C_*), Pa	Shelf Temperature (*T_S_*), °C
1	5	−10
2	10	−10
3	20	−10
4	30	−10
5	10	+10
6	30	+30

**Table 2:** Model parameters obtained by fitting the experimental data obtained with the two loading configurations.

Model Parameters	Resting Vials	Nested Vials
*A* (central), W/m^2^K	4.25	7.32
*A* (semi-border), W/m^2^K	6.08	8.62
*A* (border), W/m^2^K	11.13	15.85
*B*, W/m^2^KPa	1.90	1.50
*C*, Pa^−1^	0.04	0.06

## Data Availability

Data are available upon request to the corresponding author.

## Nomenclature

A	parameter used in Equation (3), Wm^−2^K^−1^
B	parameter used in Equation (3), Wm^−2^K^−1^Pa^−1^
C	parameter used in Equation (3), Pa^−1^
Δ*H_s_*	ice sublimation enthalpy, Jkg^−1^
H	non-dimensional position of the minimum vial temperature, -
$J_{q}$	heat flux, Wm^−2^
*K_v_*	heat transfer coefficient, Wm^−2^K^−1^
Δ*m*	water mass loss, kg
$P_{C}$	chamber pressure, Pa
S	internal cross-sectional area of the vial, m^2^
Δ*t*	ice sublimation time, s
$T_{B}$	product temperature at the vial bottom, K
$T_{S}$	shelf temperature, K
